# Factors associated with prematurity in reported cases of congenital syphilis

**DOI:** 10.11606/s1518-8787.2021055002400

**Published:** 2021-05-06

**Authors:** Maria Alix Leite Araújo, Ana Beatriz Barbosa Esteves, Ana Fátima Braga Rocha, Geraldo Bezerra da Silva, Angelica Espinosa Miranda

**Affiliations:** I Universidade de Fortaleza FortalezaCE Brasil Universidade de Fortaleza. Programa de Pós-Graduação em Saúde Coletiva. Fortaleza, CE, Brasil; II Universidade de Fortaleza FortalezaCE Brasil Universidade de Fortaleza. Programa de Pós-Graduação em Saúde Coletiva. Fortaleza, CE, Brasil; III Faculdade Terra Nordeste CaucaiaCE Brasil Faculdade Terra Nordeste. Curso de Bacharelado em Enfermagem. Caucaia, CE, Brasil; IV Universidade de Fortaleza FortalezaCE Brasil Universidade de Fortaleza. Programa de Pós-Graduação em Saúde Coletiva. Fortaleza, CE, Brasil; V Universidade Federal do Espírito Santo Departamento de Medicina Social VitoriaES Brasil Universidade Federal do Espírito Santo. Departamento de Medicina Social. Vitoria, ES, Brasil

**Keywords:** Syphilis, Congenital, Infectious Disease Transmission, Vertical, Prenatal Care, Infant, Premature, Penicillin G

## Abstract

**OBJECTIVE:**

To analyze the factors associated with prematurity in reported cases of congenital syphilis in the city of Fortaleza, Ceará, Brazil.

**METHODS:**

Cross-sectional study conducted in ten public maternity hospitals in Fortaleza, Ceará, Brazil. A total of 478 reported cases of congenital syphilis were included in 2015, and data were collected from notification forms, from mothers’ and babies’ medical records and from prenatal cards. For the bivariate analysis, Pearson’s chi-squared and Fisher’s exact tests were used, considering p < 0.05. Multiple logistic regression was conducted, presenting odds ratio (OR) with a 95% confidence interval.

**RESULTS:**

We found 15.3% prematurity in pregnant women with syphilis. The titration of the VDRL test > 1:8 at delivery (OR 2.46; 95%CI: 1.33–4.53; p = 0.004) and the non-treatment of the pregnant women or treatment with drugs other than penicillin during prenatal care (OR 3.52; 95%CI: 1.74–7.13; p< 0.001) were associated with higher chances of prematurity.

**CONCLUSION:**

The prematurity due to congenital syphilis is a preventable condition, provided that pregnant women with syphilis are treated appropriately. Weaknesses in prenatal care are associated with this outcome, which highlights the importance of public policies oriented to improve the quality of prenatal care.

## INTRODUCTION

Syphilis is a systemic infection that, when affecting the pregnant woman, can be transmitted to the baby, causing congenital syphilis (CS), with severe consequences, including prematurity^1^. The etiology of preterm delivery is multifactorial, and infectious causes, including sexually transmitted infections (STI), are relevant, given their high prevalence and association, when untreated, with undesirable obstetric outcomes^2^. In this context, syphilis takes a leading role^3^.

According to the World Health Organization (WHO), in 2016, about 1 million pregnant women were infected with syphilis, which had the consequence of children with early and late clinical manifestations of CS^4^. In the Americas, the incidence rate of CS has been increasing over the years, and the reported Brazilian cases have contributed considerably^5^.

In Brazil, in 2018 alone, reported cases included 158,051 of acquired syphilis, 62,599 of syphilis in pregnant women, and 26,219 of CS. The state of Ceará is among those with the highest rates^6^, and its capital, Fortaleza, reported in that same year 827 cases of CS^7^, with an incidence rate always increasing over the years^8^.

Considering the severity of outcomes, control measures have been recommended^9^. The reduction of CS is contemplated in the Sustainable Development Goals, which seek to eliminate preventable deaths of newborns and children under 5 years, reduce neonatal mortality and fight communicable infections^10^. In this context, prematurity is highlighted, since it is the leading cause of death in children under 5 years in the world^11^^,^^12^, contributing to increase neonatal mortality in poor and developing countries^13^.

In view of the aforementioned aspects and considering the relevant and persistent gaps in studies on prematurity by CS, this paper seeks to analyze the factors associated with prematurity in CS reported cases in Fortaleza, Ceará, Brazil.

## METHODS

This cross-sectional study, which analyzed the prevalence and the factors associated with prematurity in pregnant women with syphilis, used data from all ten public maternity hospitals in Fortaleza, capital of Ceará, Brazil.

The city has about 2.5 million inhabitants, gross domestic product of R$ 41.2 billion, and human development index of 0.754^14^. The coverage of the Brazilian Family Health Strategy (FHS) is 57.6%, and the coverage of prenatal care is 95% (unpublished data, provided by the Municipal Health Department).

All CS reported cases in 2015 were included in the study, considering children who were born alive, i.e., deliveries in which there was expulsion or complete extraction of the fetus, regardless of the mother’s gestational age, of the product of conception and of any sign of life (heartbeat, breathing or even umbilical cord pulsation) after maternal separation. To define CS, the following criteria were considered, established by the Brazilian Ministry of Health^15^ (p. 26):

- Child whose mother had, during prenatal care or at delivery, reactive (with any titration) non-treponemal tests and reactive treponemal test, and who has not been treated or has received inadequate treatment.
- Child whose mother was not diagnosed with syphilis during pregnancy and, given the impossibility of the maternity hospital to take the treponemal test on her, presented reactive (with any titration) non-treponemal test at delivery.
- Child whose mother was not diagnosed with syphilis during pregnancy and, given the impossibility of the maternity hospital to take the treponemal test on her, presented reactive treponemal test at delivery.
- Child whose mother presented reactive treponemal test and non-reactive non-treponemal test at the time of delivery, without prior treatment.

Cases of abortions and stillbirths were excluded, due to the unavailability of information for many of the variables included in this study, which would impair the analysis. In addition, were also excluded the cases of ectopic pregnancy, molar pregnancy or abortion, and cases in which pregnant women had heart diseases, severe lung diseases, severe nephropathies, endocrinopathies, hematological diseases and chronic arterial hypertension, neurological diseases, psychiatric diseases requiring follow-up, autoimmune diseases, genetic alterations, history of deep vein thrombosis or pulmonary embolism, gynecological diseases, Hansen’s disease, tuberculosis or any clinical pathology requiring specialized follow-up, as well as cases of patients using antihypertensive (BP > 140/90mmHg before 20 weeks of gestational age – GI). The exclusion of these diseases occurred because they were related to prematurity risk^6^^–^^8^, possibly acting as a confounding factor for prematurity by CS.

Data were collected between June and September 2018. All cases of CS reported in 2015 in the *Sistema de Informação de Agravos de Notificação* (SINAN – Notifiable Diseases Information System) were surveyed. Data were collected from the notification forms and, later, from the medical records of the mother and baby and from the pregnant woman prenatal card. It is noteworthy that maternity hospitals attach a copy of the card to the medical record. When there were divergences between the sources, the data from the medical records were considered valid.

The analyzed outcome was prematurity (when the child is born with less than 37 full weeks of gestation). The following maternal variables were analyzed: sociodemographic (age, marital status, education, occupation) and related to prenatal care (attendance to prenatal care, number of consultations, beginning of follow-up, examinations for syphilis, number of tests for syphilis, results of the first and second tests for syphilis, titration of the VDRL test in prenatal care, time of syphilis diagnosis, treatment in prenatal care, treatment of the partner and titration of the VDRL test at delivery) and use of illicit drugs.

Data were analyzed in the statistical software SPSS (Statistical Package for the Social Sciences, version 23, IBM, USA) and in the Stata, version 10.0 (Stata Corp., USA), by Stepwise method for multiple logistic regression. For the bivariate analysis, Pearson’s chi-squared and Fisher’s exact tests were used, considering p < 0.05. The adjusted analysis included the variables with p < 0.20, remaining those with p < 0.05. As a measure of effect, the odds ratio (OR) and the 95% confidence interval were used.

This study was approved by the Research Ethics Committee of the Universidade de Fortaleza (Unifor), Opinion No. 2,110,189, and is part of a larger study entitled *Manifestações Clínicas e Complicações em Crianças com Sífilis Congênita* (Clinical Manifestations and Complications in Children with Congenital Syphilis).

## RESULTS

In 2015, 674 cases of CS were reported in Fortaleza, of which 478 were considered eligible. We excluded 196 cases (31 abortions, 39 stillborns and 126 pregnant women who presented other risk problems for prematurity). A total of 73 children (15.3%) were born premature.

Table 1 shows the sociodemographic and obstetric profile of the pregnant women with syphilis who had premature babies. The mean age was 24.9 years (SD = 6.3), and most were between 20 and 29; 54 (74%) completed elementary school and 26 (35.6%) reported the use of some illicit drug. A number of 52 women attended prenatal care (71.2%); of these, 37 (71.1%) were tested at least once for syphilis; and 11 (21.2%) underwent treatment with at least one dose of benzathine penicillin. At delivery, 32 (43.8%) presented titration > 1:8 on the VDRL test (Table 1).

**Table 1:** Sociodemographic and obstetric profile of pregnant women with syphilis and prematurity outcome (Fortaleza, Ceará, 2015).

Variables		n^a^	%
Age (in years)			
	≤ 19	15	20.5
	20–29	41	56.2
	≥ 30	17	23.3
Marital status			
	With steady partner	36	49.3
	Without steady partner	37	50.7
Education level (years of study)			
	Complete elementary school	54	74.0
	Incomplete high school / complete high school / higher education	19	26.0
Has paid work			
	Yes	13	17.8
	No	60	82.2
Illicit drug user			
	Yes	26	35.6
	Not/ignored	47	64.4
Attended prenatal care			
	Yes	52	71.2
	No	21	28.8
Number of prenatal consultations			
	1	8	15.4
	2 to 5	26	50.0
	≥ 6	18	34.6
Onset of prenatal care			
	First trimester	23	44.2
	Second trimester	20	38.5
	Third trimester	9	17.3
Number of tests for syphilis in prenatal care^b^			
	None	15	28.9
	1	31	59.6
	2	6	11.5
Prenatal syphilis test result^b^			
	Reactive	21	56.8
	Nonreactive	16	43.2
VDRL titration in prenatal care^c^			
	≤ 1:8	4	30.8
	> 1:8	9	69.2
Treatment during prenatal care			
	Complete treatment^d^	4	7.7
	Incomplete treatment^d^	7	13.5
	Other drug/Untreated	41	78.8
VDRL titration at delivery			
	≤ 1:8	41	56.2
	> 1:8	32	43.8

a Not all patients underwent prenatal care or all tests for syphilis, so the “n” of some variables is different.

b Rapid test or VDRL test.

c Only for those who underwent VDRL test.

d Treatment with three doses of benzathine penicillin (7.2 million IU) regardless of partner treatment.

Table 2 describes the data from pregnant women whose VDRL titration was > 1:8 at delivery. Among pregnant women with syphilis who had premature babies, 24 (75%) attended prenatal care; of these, 9 (37.5%) were never tested for syphilis. All those with reactive results in prenatal care had titration > 1:8. The cases of prematurity occurred in pregnant women who underwent incomplete treatment and, mainly, who were not treated or received some drug other than penicillin.

**Table 2:** Analysis of prenatal care of pregnant women who had VDRL titration > 1:8 at delivery (Fortaleza, Ceará, 2015).

Variables		VDRL delivery > 1:8			
		Total cases of congenital syphilis (n = 134)		Prematurity (n = 32)	
Attended prenatal care					
	Yes	111	82.8	24	75.0
	No	23	17.2	8	25.0
Number of prenatal consultations					
	1	8	7.2	5	20.8
	2 to 5	53	47.7	13	54.2
	≥ 6	50	45.0	6	25.0
Onset of prenatal care					
	First trimester	49	44.2	8	33.3
	Second trimester	45	40.5	10	41.7
	Third trimester	17	15.3	6	25.0
Number of tests for syphilis in prenatal care^b^					
	None	28	25.2	9	37.5
	1	65	58.6	13	54.2
	2	18	16.2	2	8.3
Prenatal syphilis test result^b^					
	Reactive	65	78.3	6	40.0
	Nonreactive	18	21.7	9	60.0
VDRL titration in prenatal care^c^					
	≤ 1:8	13	23.6	–	–
	> 1:8	42	76.4	6	100.0
Treatment during prenatal care					
	Complete treatmen^t^d	24	21.6	–	–
	Incomplete treatment^d^	22	19.8	3	12.5
	Other drug/Untreated	65	58.6	21	87.5

a Not all patients underwent prenatal care or all tests for syphilis, so the “n” of some variables is different.

b Rapid test or VDRL test.

c Only for those who underwent VDRL test.

d Treatment with three doses of benzathine penicillin (7.2 million IU) regardless of partner treatment.

In the bivariate analysis, the following situations were associated with the outcome of prematurity: illicit drug use (p = 0.001; OR: 2.39; 95%CI: 1.39–4.11), not having attended prenatal care (p = 0.004; OR: 2.28; 95%CI: 1.28–4.05), having been diagnosed with syphilis at delivery (p < 0.001; OR: 3.29; 95%CI: 1.93–5.61), and having presented VDRL titration at delivery > 1:8 (p = 0.001; OR: 2.31; 95%CI: 1.38–3.87) (Table 3).

**Table 3:** Sociodemographic, prenatal and delivery aspects of pregnant women with syphilis associated with the outcome of prematurity (Fortaleza, Ceará, 2015).

Variables		n (%)	Prematurity				p	Crude OR (95%CI)
			Yes		No			
			n	%	n	%		
Age (in years)							0.484	
	≥ 19	405 (84.7)	60	14.8	345	85.2		1
	≤ 18	73 (15.3)	13	17.8	60	82.2		1.25 (0.64–2.41)
Marital status							0.250	
	With steady partner	267 (55.8)	36	13.5	231	86.5		1
	Without steady partner	211 (44.2)	37	17.5	174	82.5		1.36 (0.83–2.25)
Education level (years of study)							0.410	
	Incomplete high school / complete high school / higher education	145 (30.3)	19	13.1	126	86.9		1
	Complete elementary school	333 (69.7)	54	16.2	279	83.8		1.28 (0.73–2.25)
Has paid work							0.060	
	Yes	128 (26.8)	13	10.2	115	89.8		1
	No	350 (73.2)	60	17.1	290	82.9		1.83 (0.97–3.46)
Illicit drug user							0.001	
	Not/ignored	376 (78.7)	47	12.5	329	87.5		1
	Yes	102 (21.3)	26	25.5	76	74.5		2.39 (1.39–4.11)
Attended prenatal care							0.004	
	Yes	396 (82.8)	52	13.1	344	86.9		1
	No	82 (17.2)	21	25.6	61	74.4		2.28 (1.28–4.05)
Time of syphilis diagnosis							< 0.001	
	Prenatal care	267 (55.9)	23	8.6	244	91.4		1
	Delivery	211 (44.1)	50	23.7	161	76.3		3.29 (1.93–5.61)
VDRL titration at delivery							0.001	
	≤ 1:8	344 (72.0)	41	11.9	303	88.1		1
	> 1:8	134 (28.0)	32	23.9	102	76.1		2.31 (1.38–3.87)

When analyzing only pregnant women who attended prenatal care, prematurity was associated with: having attended less than six consultations (p = 0.005; OR: 2.35%; 95%CI: 1.28–4.33), never been tested for syphilis (p = 0.008; OR: 2.44%; 95%CI: 1.24–4.77), having not been treated or having received treatment with some drug other than benzathine penicillin (p < 0.001; OR: 3.59; 95%CI:1.79–7.23) (Table 4).

**Table 4:** Prenatal care of pregnant women with syphilis associated with the outcome of prematurity (Fortaleza, Ceará, 2015).

Variables		n (%)	Prematurity				p	Crude OR (95%CI)
			Yes		No			
			n	%	n	%		
Number of consultations							0.005	
	≥ 6	209 (52.8)	18	8.6	191	91.4		1
	< 6	187 (47.2)	34	18.2	153	81.8		2.35 (1.28–4.33)
Onset of prenatal care							0.092	
	First and second trimesters	354 (89.4)	43	12.1	311	87.9		1
	Third trimester	42 (10.6)	9	21.4	33	78.6		1.97 (0.88–4.40)
Number of tests for syphilis in prenatal care							0.008	
	At least one	332 (83.8)	37	11.1	295	88.9		1
	None	64 (16.2)	15	23.4	49	76.6		2.44 (1.24–4.77)
Underwent two VDRL tests in prenatal care							0.112	
	Yes	78 (19.7)	6	7.7	72	92.3		1
	Not/ignored	318 (80.3)	46	14.5	272	85.5		2.02 (0.83–4.93)
Treatment of the pregnant women in prenatal care							< 0.001	
	With benzathine penicillin	180 (45.4)	11	6.1	169	93.9		1
	Other drug/Untreated	216 (54.6)	41	19.0	175	81.0		3.59 (1.79–7.23)

Table 5 shows the analysis of multiple logistic regression, adjusted, of sociodemographic, prenatal care and deliveries aspects of pregnant women with syphilis associated with the outcome of prematurity. We found that women who had VDRL titration > 1:8 at delivery and those who were not treated or received treatment with some drug other than penicillin were 2.46 (95%CI: 1.33–4.53; p = 0.004) and 3.52 (95%CI: 1.74–7.13; p< 0.001) times more likely to have premature babies, respectively.

**Table 5:** Adjusted multiple logistic regression analysis of sociodemographic, prenatal care and delivery aspects of pregnant women with syphilis associated with the outcome of prematurity (Fortaleza, Ceará, 2015).

Variables		n (%)	Adjusted OR (95%CI)	p
VDRL titration at delivery				0.004
	≤ 1:8	344 (72.0)	1	
	> 1:8	134 (28.0)	2.46 (1.33–4.53)	
Treatment of pregnant women in prenatal care				< 0.001
	With benzathine penicillin	180 (45.4)	1	
	Other drug/Untreated	216 (54.6)	3.52 (1.74–7.13)	

## DISCUSSION

In this study, we found the outcome of prematurity in 15.3% of the cases of CS, a percentage higher than the estimated rates for pregnant women in general, described in a Brazilian study: between 7.7%^16^ and 11.1%^17^. Pregnant women who were not treated or treated with drugs other than benzathine penicillin during prenatal care, as well as those who had high VDRL titers at delivery, had more outcomes of prematurity.

We can observe that a significant number of pregnant women with syphilis who had premature babies attended prenatal care. However, many opportunities to prevent CS were lost, a situation also evidenced in another study conducted in six Brazilian capitals^18^. Prenatal care can positively impact the health of pregnant women and avoid infant mortality^19^, and it is an important predictor to prevent unfavorable outcomes related to syphilis during pregnancy, provided it is conducted with quality^20^^,^^21^.

Among the lost opportunities, we highlight the lack of diagnosis and failures in the treatment of pregnant women. A meta-analysis study showed that prenatal interventions significantly reduce the risk of pregnant women having an adverse result due to syphilis^22^. However, during the period of this study, the routine examination requested to the pregnant women was the VDRL test, there was no daily blood collection in the units, and the results were available only 15 days after collection, hindering the diagnosis. In addition, the rapid testing (RT) was being implemented.

The RT for syphilis is a strategy that can increase the coverage of testing in pregnant women, especially when conducted at the first consultation, enabling immediate treatment. Considering that most pregnant women attended two or more consultations and started prenatal care before the end of the second trimester of pregnancy, there would be time for adequate treatment if they had undergone RT.

However, during the period of this study, Brazil experienced a severe penicillin scarcity^23^, contributing to many pregnant women not being treated or receiving drugs other than benzathine penicillin. These pregnant women were 3.52 times more likely to have premature babies, a situation also evidenced in studies conducted in China, which identified higher chances of prematurity and other outcomes in pregnant women with untreated or inadequately treated syphilis^24^^,^^25^.

The possibility of a new penicillin scarcity^26^ is worrisome, since benzathine penicillin is the drug of choice for the treatment of syphilis in pregnant women, as it is the only one that crosses the transplacental barrier and treats the baby^23^. For this reason, it is necessary to develop studies evaluating the efficacy of alternative drugs^27^.

In this study, we also identified a higher chance of prematurity in pregnant women whose VDRL titration was > 1:8 at delivery. High VDRL titers in pregnant women during prenatal care and at delivery may represent active syphilis, a condition associated with severe CS outcomes, including prematurity^3^^,^^28^. For this reason, after treatment, the cure control of syphilis in pregnant women should occur monthly, by monitoring the decrease of VDRL titers^15^.

A detailed analysis of the cases of pregnant women who had high VDRL titers at delivery showed that no woman who received complete treatment (three doses of benzathine penicillin) had a premature baby, reinforcing the importance of timely diagnosing and treating pregnant women with syphilis, thus preventing serious outcomes, such as prematurity due to CS.

This study has, as limitation, the analysis of secondary data, considering that lack of registration or low-quality data are common. Including the collection in different data sources (notification forms and medical records) contributed greatly to minimize the problem. Moreover, the exclusion of stillborns, due to the lack of registration of important variables, and the exclusion of pregnant women who presented injuries that could interfere in the outcome of prematurity by CS, may have underestimated the number of premature infants.

The findings of this study show that prematurity due to CS is preventable, provided that pregnant women with syphilis are treated appropriately. We also identified that weaknesses in prenatal care are associated with this outcome, which highlights the importance of implementing public policies to improve the quality of prenatal care.

In this sense, primary health care has an indispensable role to detect syphilis in pregnant women and ensure the testing and treatment. These efforts to prevent prematurity by CS can avoid severe consequences for the baby, the family and the health system.
