# Supplementary material for: Factors associated with prematurity in reported cases of congenital syphilis
Source: Rev Saude Publica. 2021 May 6;55:28. doi: 10.11606/s1518-8787.2021055002400 (PMC8102023; doi:10.11606/s1518-8787.2021055002400)
Supplement: Translation

# Translation: Fatores associados à prematuridade em casos notificados de sífilis congênita

**Authors:** Maria Alix Leite Araújo, Ana Beatriz Barbosa Esteves, Ana Fátima Braga Rocha, Geraldo Bezerra da Silva, Angelica Espinosa Miranda

**Affiliations:** I FortalezaCE Brasil Universidade de Fortaleza. Programa de Pós-Graduação em Saúde Coletiva. Fortaleza, CE, Brasil; II FortalezaCE Brasil Universidade de Fortaleza. Programa de Pós-Graduação em Saúde Coletiva. Fortaleza, CE, Brasil; III CaucaiaCE Brasil Faculdade Terra Nordeste. Curso de Bacharelado em Enfermagem. Caucaia, CE, Brasil; IV FortalezaCE Brasil Universidade de Fortaleza. Programa de Pós-Graduação em Saúde Coletiva. Fortaleza, CE, Brasil; V VitoriaES Brasil Universidade Federal do Espírito Santo. Departamento de Medicina Social. Vitoria, ES, Brasil

**Keywords:** Sífilis Congênita, Transmissão Vertical de Doença Infecciosa, Cuidado Pré-Natal, Recém-Nascido Prematuro, Penicilina G

## Abstract

**OBJETIVO:**

Analisar os fatores associados à prematuridade em casos notificados de sífilis congênita no município de Fortaleza, Ceará, Brasil.

**MÉTODOS:**

Estudo transversal realizado em dez maternidades públicas de Fortaleza, Ceará, Brasil. Foram incluídos 478 casos notificados de sífilis congênita em 2015, e os dados foram coletados das fichas de notificação, dos prontuários das mães e dos bebês e do cartão de pré-natal. Para a análise bivariada, foram utilizados os testes do qui-quadrado de Pearson e exato de Fisher, considerando p < 0,05. Realizou-se regressão logística múltipla, apresentando razão de chances (OR) com intervalo de confiança de 95%.

**RESULTADOS:**

Encontrou-se 15,3% de prematuridade em gestantes com sífilis. A titulação do teste VDRL > 1:8 no parto (OR 2,46; IC95%: 1,33–4,53; p = 0,004), o não tratamento da gestante ou tratamento realizado com drogas diferentes da penicilina durante o pré-natal (OR 3,52; IC95%: 1,74–7,13; p < 0,001) estiveram associados a maiores chances de prematuridade.

**CONCLUSÃO:**

A prematuridade decorrente da sífilis congênita é um agravo evitável, desde que as gestantes com sífilis sejam tratadas adequadamente. As fragilidades na assistência pré-natal estão associadas a este desfecho, o que ressalta a importância de implementar políticas públicas voltadas a melhorar a qualidade do pré-natal.

## INTRODUÇÃO

A sífilis é uma infecção sistêmica que, quando acomete a gestante, pode ser transmitida ao bebê, causando a sífilis congênita (SC), com consequências graves, dentre elas a prematuridade^1^. A etiologia do parto prematuro é multifatorial, e as causas infecciosas, dentre elas as infecções sexualmente transmissíveis (IST), são relevantes, tendo em vista sua alta prevalência e associação, quando não tratadas, a desfechos obstétricos indesejáveis^2^. Neste contexto, a sífilis assume protagonismo^3^.

Segundo a Organização Mundial da Saúde (OMS), em 2016, cerca de 1 milhão de gestantes estavam infectadas com sífilis, o que teve como consequência crianças com manifestações clínicas precoces e tardias da SC^4^. Nas Américas, a taxa de incidência de SC vem crescendo ao longo dos anos, e os casos notificados no Brasil contribuíram consideravelmente para esse aumento^5^.

No Brasil, somente em 2018, foram notificados 158.051 casos de sífilis adquirida, 62.599 casos de sífilis em gestantes, e 26.219 de SC. O estado do Ceará está dentre aqueles que apresentaram as maiores taxas do país^6^, e sua capital, Fortaleza, notificou nesse mesmo ano 827 casos de SC^7^, com taxa de incidência sempre crescente ao longo dos anos^8^.

Considerando a gravidade dos desfechos, medidas de controle têm sido recomendadas^9^. A redução da SC está contemplada nos Objetivos para o Desenvolvimento Sustentável, que buscam eliminar as mortes evitáveis de recém-nascidos e crianças menores de 5 anos, reduzir a mortalidade neonatal e combater as infecções transmissíveis^10^. Neste contexto, a prematuridade tem destaque, considerando que ela é a principal causa de morte em crianças menores de 5 anos no mundo^11^^,^^12^, contribuindo para aumentar a mortalidade neonatal em países pobres e em desenvolvimento^13^.

Diante dos aspectos supracitados, e considerando que ainda persistem lacunas importantes nos estudos acerca da prematuridade por SC, este artigo tem por objetivo analisar os fatores associados à prematuridade em casos notificados de SC no município de Fortaleza, Ceará, Brasil.

## MÉTODOS

Este estudo transversal, que analisou a prevalência e os fatores associados ao desfecho de prematuridade em gestantes com sífilis, utilizou dados de todas as dez maternidades públicas do município de Fortaleza, capital do estado do Ceará.

Fortaleza tem população de cerca de 2,5 milhões de habitantes, produto interno bruto de R$ 41,2 bilhões, e índice de desenvolvimento humano de 0,754^14^. A cobertura da Estratégia Saúde da Família é de 57,6%, e a de pré-natal de 95% (dados não publicados, disponibilizados pela Secretaria Municipal de Saúde).

Fizeram parte do estudo todos os casos notificados de SC em 2015, considerando crianças que nasceram vivas, isto é, partos em que houve expulsão ou extração completa do feto, independentemente da idade gestacional da mãe, do produto da concepção e da apresentação, depois da separação materna, de qualquer sinal de vida (batimentos cardíacos, respiração ou até mesmo pulsação do cordão umbilical). Para definir a SC, foram considerados os seguintes critérios, estabelecidos pelo Ministério da Saúde^15^(p. 26):

- Criança cuja mãe apresente, durante o pré-natal ou no momento do parto, testes para sífilis não treponêmico reagente com qualquer titulação e treponêmico reagente, e que não tenha sido tratada ou tenha recebido tratamento inadequado.
- Criança cuja mãe não foi diagnosticada com sífilis durante a gestação e, na impossibilidade de a maternidade realizar o teste treponêmico, apresente teste não treponêmico reagente com qualquer titulação no momento do parto.
- Criança cuja mãe não foi diagnosticada com sífilis durante a gestação e, na impossibilidade de a maternidade realizar o teste não treponêmico, apresente teste treponêmico reagente no momento do parto.
- Criança cuja mãe apresente teste treponêmico reagente e teste não treponêmico não reagente no momento do parto, sem registro de tratamento prévio.

Foram excluídos os casos de abortos e natimortos, devido à indisponibilidade de informações para muitas das variáveis inseridas neste estudo, o que prejudicaria a análise. Além disso, também foram excluídos: casos de gravidez ectópica, mola hidatiforme ou aborto, e casos em que as gestantes apresentaram cardiopatias, pneumopatias graves, nefropatias graves, endocrinopatias, doenças hematológicas e hipertensão arterial crônica, doenças neurológicas, doenças psiquiátricas que necessitam de acompanhamento, doenças autoimunes, alterações genéticas, antecedente de trombose venosa profunda ou embolia pulmonar, doenças ginecológicas, hanseníase, tuberculose ou qualquer patologia clínica que necessite de acompanhamento especializado, bem como casos de pacientes que fazem uso de anti-hipertensivo (PA > 140/90mmHg antes de 20 semanas de idade gestacional – IG). A exclusão desses agravos ocorreu por estarem relacionadas a risco de prematuridade^6^^–^^8^, podendo atuar como fator de confundimento para prematuridade por SC.

A coleta de dados foi realizada entre os meses de janeiro e novembro de 2018. Foram levantados todos os casos de SC notificados em 2015 no Sistema de Informação de Agravos de Notificação (Sinan). Os dados foram colhidos das fichas de notificação e, posteriormente, dos prontuários médicos da mãe e do bebê e do cartão de pré-natal da gestante. Vale salientar que as maternidades anexam uma cópia do cartão da gestante ao prontuário. Quando havia divergências entre as fontes de dados, foram considerados válidos os dados dos prontuários.

O desfecho analisado foi prematuridade (quando a criança nasce com menos de 37 semanas completas de gestação). Analisaram-se as seguintes variáveis maternas: sociodemográficas (idade, situação conjugal, escolaridade, ocupação) e relacionadas à assistência pré-natal (frequentou o pré-natal, número de consultas, início do acompanhamento, realização dos exames para a sífilis, número de exames para sífilis, resultado do primeiro e segundo exames para sífilis, titulação do teste VDRL no pré-natal, momento do diagnóstico de sífilis, tratamento da gestante no pré-natal, tratamento do parceiro e titulação do teste VDRL no parto) e uso de drogas ilícitas.

Os dados foram analisados no programa estatístico SPSS (Statistical Package for the Social Sciences, versão 23, IBM, USA) e no Stata versão 10.0 *(Stata Corp., USA)*, pela técnica de *Stepwise* para regressão logística múltipla. Para a análise bivariada, utilizou-se o teste qui-quadrado de Pearson e teste exato de Fisher, considerando p < 0,05. A análise ajustada incluiu as variáveis com valor de p < 0,20, permanecendo as que tiveram valor de p < 0,05. Como medida de efeito, utilizou-se a razão de chances (OR) e o intervalo de confiança de 95%.

Este estudo foi aprovado pelo Comitê de Ética e Pesquisa da Universidade de Fortaleza (Unifor), sob o Parecer nº 2.110.189, e faz parte de uma pesquisa maior, intitulada “Manifestações Clínicas e Complicações em Crianças com Sífilis Congênita”.

## RESULTADOS

No ano de 2015, foram notificados 674 casos de SC em Fortaleza, dos quais 478 foram considerados elegíveis. Excluíram-se 196 casos (31 abortos, 39 natimortos e 126 gestantes que apresentaram outros agravos de risco para prematuridade). Nasceram prematuras 73 crianças (15,3%).

O perfil sociodemográfico e obstétrico das gestantes com sífilis que tiveram bebês prematuros está apresentado na Tabela 1. A média de idade das gestantes foi de 24,9 anos (DP = 6,3), e a maioria tinha entre 20 e 29 anos de idade; 54 (74%) completaram o ensino fundamental e 26 (35,6%) referiram uso de alguma droga ilícita. Cinquenta e duas mulheres frequentaram o pré-natal (71,2%); destas, 37 (71,1%) realizaram pelo menos um exame para sífilis; e 11 (21,2%) realizaram tratamento com pelo menos uma dose de penicilina benzatina. Na ocasião do parto, 32 (43,8%) apresentaram titulação > 1:8 no exame de VDRL (Tabela 1).

**Tabela 1:** Perfil sociodemográfico e obstétrico de gestantes com sífilis com desfecho de prematuridade (Fortaleza, Ceará, 2015).

Variáveis		n^a^	%
Idade (em anos)			
	≤ 19	15	20,5
	20–29	41	56,2
	≥ 30	17	23,3
Situação conjugal			
	Com parceiro fixo	36	49,3
	Sem parceiro fixo	37	50,7
Escolaridade (anos de estudo)			
	Até ensino fundamental completo	54	74,0
	Ensino médio incompleto/completo/superior	19	26,0
Possui trabalho remunerado			
	Sim	13	17,8
	Não	60	82,2
Usuária de drogas ilícitas			
	Sim	26	35,6
	Não/ignorado	47	64,4
Frequentou pré-natal			
	Sim	52	71,2
	Não	21	28,8
	Número de consultas no pré-natal		
	1	8	15,4
	2 a 5	26	50,0
	≥ 6	18	34,6
Início do pré-natal			
	1º trimestre	23	44,2
	2º trimestre	20	38,5
	3º trimestre	9	17,3
Número de exames para sífilis no pré-natal^b^			
	Nenhum	15	28,9
	1	31	59,6
	2	6	11,5
Resultado do exame para sífilis no pré-natal^b^			
	Reagente	21	56,8
	Não reagente	16	43,2
Titulação do VDRL no pré-natal^c^			
	≤ 1:8	4	30,8
	> 1:8	9	69,2
	Tratamento realizado no pré-natal		
	Tratamento completo^d^	4	7,7
	Tratamento incompleto	7	13,5
	Outra droga/Não tratada	41	78,8
Titulação do VDRL do parto			
	≤ 1:8	41	56,2
	> 1:8	32	43,8

a Nem todas as pacientes realizaram pré-natal ou todos os exames para sífilis, por isso o “n” de algumas variáveis é diferente.

b Teste rápido ou exame de VDRL.

c Apenas para aquelas que realizaram exame de VDRL.

d Tratamento com três doses de penicilina benzatina (7,2 milhões UI) independente do tratamento do parceiro.

Os dados de gestantes cuja titulação do VDRL foi > 1:8 no momento do parto estão descritos na Tabela 2. Dentre as gestantes com sífilis que tiveram bebês prematuros, 24 (75%) frequentaram o pré-natal; destas, 9 (37,5%) não realizaram nenhum exame para sífilis. Todas que apresentaram resultado reagente no pré-natal tiveram titulação > 1:8. Os casos de prematuridade ocorreram em gestantes que realizaram tratamento incompleto e, principalmente, que não foram tratadas ou receberam outra droga diferente da penicilina.

**Tabela 2:** Análise da assistência pré-natal de gestantes que apresentaram titulação de VDRL > 1:8 no momento do parto (Fortaleza, Ceará, 2015).

Variáveis		VDRL parto > 1:8			
		Total de casos sífilis congênita (n = 134)		Prematuridade (n = 32)	
		n^a^	%	n^a^	%
Frequentou pré-natal					
	Sim	111	82,8	24	75,0
	Não	23	17,2	8	25,0
Número de consultas no pré-natal					
	1	8	7,2	5	20,8
	2 a 5	53	47,7	13	54,2
	≥ 6	50	45,0	6	25,0
Início do pré-natal					
	1º trimestre	49	44,2	8	33,3
	2º trimestre	45	40,5	10	41,7
	3º trimestre	17	15,3	6	25,0
Número de exames para sífilis no pré-natal^b^					
	Nenhum	28	25,2	9	37,5
	1	65	58,6	13	54,2
	2	18	16,2	2	8,3
Resultado do exame para sífilis no pré-natal^b^					
	Reagente	65	78,3	6	40,0
	Não reagente	18	21,7	9	60,0
Titulação do VDRL no pré-natal^c^					
	≤ 1:8	13	23,6	–	–
	> 1:8	42	76,4	6	100,0
	Tratamento realizado no pré-natal				
	Tratamento completo^d^	24	21,6	–	–
	Tratamento incompleto	22	19,8	3	12,5
	Outra droga/não tratada	65	58,6	21	87,5

a Nem todas as pacientes realizaram pré-natal ou todos os exames para sífilis, por isso o “n” de algumas variáveis é diferente.

b Teste rápido e/ou exame de VDRL.

c Apenas para aquelas que realizaram exame de VDRL.

d Tratamento com três doses de penicilina benzatina (7,2 milhões UI), independente do tratamento do parceiro.

Na análise bivariada, mostraram associação com o desfecho de prematuridade: uso de droga ilícita (p = 0,001; OR: 2,39; IC95%: 1,39–4,11), não ter frequentado o pré-natal (p = 0,004; OR: 2,28; IC95%: 1,28–4,05), ter sido diagnosticada com sífilis no momento do parto (p < 0,001; OR: 3,29; IC95%: 1,93–5,61), ter apresentado titulação do VDRL do parto > 1:8 (p = 0,001; OR: 2,31; IC95%: 1,38–3,87) (Tabela 3).

**Tabela 3:** Aspectos sociodemográficos, do pré-natal e do parto de gestantes com sífilis associados ao desfecho de prematuridade (Fortaleza, Ceará, 2015).

Variáveis		n (%)	Prematuridade				p	OR bruto (IC95%)
			Sim		Não			
			n	%	n	%		
Idade (em anos)							0,484	
	≥ 19	405 (84,7)	60	14,8	345	85,2		1
	≤ 18	73 (15,3)	13	17,8	60	82,2		1,25 (0,64–2,41)
Situação conjugal							0,250	
	Com parceiro fixo	267 (55,8)	36	13,5	231	86,5		1
	Sem parceiro fixo	211 (44,2)	37	17,5	174	82,5		1,36 (0,83–2,25)
Escolaridade (anos de estudo)							0,410	
	Ensino médio incompleto/completo/superior	145 (30,3)	19	13,1	126	86,9		1
	Até ensino fundamental completo	333 (69,7)	54	16,2	279	83,8		1,28 (0,73–2,25)
Possui trabalho remunerado							0,060	
	Sim	128 (26,8)	13	10,2	115	89,8		1
	Não	350 (73,2)	60	17,1	290	82,9		1,83 (0,97–3,46)
Usuária de drogas ilícitas							0,001	
	Não/ignorado	376 (78,7)	47	12,5	329	87,5		1
	Sim	102 (21,3)	26	25,5	76	74,5		2,39 (1,39–4,11)
Frequentou pré-natal							0,004	
	Sim	396 (82,8)	52	13,1	344	86,9		1
	Não	82 (17,2)	21	25,6	61	74,4		2,28 (1,28–4,05)
Momento do diagnóstico de sífilis							< 0,001	
	Pré-natal	267 (55,9)	23	8,6	244	91,4		1
	Parto	211 (44,1)	50	23,7	161	76,3		3,29 (1,93–5,61)
Titulação do VDRL do parto							0,001	
	≤ 1:8	344 (72,0)	41	11,9	303	88,1		1
	> 1:8	134 (28,0)	32	23,9	102	76,1		2,31 (1,38–3,87)

Ao analisar somente as gestantes que frequentaram o pré-natal, a prematuridade esteve associada a: ter recebido menos de seis consultas (p = 0,005; OR: 2,35; IC 95%: 1,28–4,33), não ter realizado nenhum exame para sífilis (p = 0,008; OR: 2,44; IC 95%: 1,24–4,77), não ter sido tratada ou ter recebido tratamento com droga diferente da penicilina benzatina (p < 0,001; OR: 3,59; IC 95%:1,79–7,23) (Tabela 4).

**Tabela 4:** Assistência pré-natal de gestantes com sífilis associada ao desfecho de prematuridade (Fortaleza, Ceará, 2015).

Variáveis		n (%)	Prematuridade				p	OR bruto (IC95%)
			Sim		Não			
			n	%	n	%		
Número de consultas							0,005	
	≥ 6	209 (52,8)	18	8,6	191	91,4		1
	< 6	187 (47,2)	34	18,2	153	81,8		2,35 (1,28–4,33)
Início do pré-natal							0,092	
	1º e 2º trimestres	354 (89,4)	43	12,1	311	87,9		1
	3º trimestre	42 (10,6)	9	21,4	33	78,6		1,97 (0,88–4,40)
Número de exames para sífilis no pré-natal							0,008	
	Pelo menos um	332 (83,8)	37	11,1	295	88,9		1
	Nenhum	64 (16,2)	15	23,4	49	76,6		2,44 (1,24–4,77)
Realizou dois VDRL no pré-natal							0,112	
	Sim	78 (19,7)	6	7,7	72	92,3		1
	Não/Ignorado	318 (80,3)	46	14,5	272	85,5		2,02 (0,83–4,93)
Tratamento da gestante no pré-natal							< 0,001	
	Com penicilina benzatina	180 (45,4)	11	6,1	169	93,9		1
	Outra droga/não tratada	216 (54,6)	41	19,0	175	81,0		3,59 (1,79–7,23)

Na Tabela 5, é apresentada a análise de regressão logística múltipla, ajustada, dos aspectos sociodemográficos, da atenção pré-natal e partos de gestantes com sífilis associados ao desfecho de prematuridade. Encontrou-se que mulheres que apresentaram titulação do VDRL > 1:8 na ocasião do parto e aquelas que não foram tratadas ou receberam tratamento com droga diferente da penicilina apresentaram 2,46 (IC95%: 1,33–4,53; p = 0,004) e 3,52 (IC95%: 1,74–7,13; p < 0,001) vezes mais chance de ter bebês prematuros, respectivamente.

**Tabela 5:** Análise de regressão logística múltipla ajustada dos aspectos sociodemográficos, da atenção pré-natal e parto de gestantes com sífilis associados ao desfecho de prematuridade (Fortaleza, Ceará, 2015).

Variáveis		n (%)	OR ajustado (IC95%)	p
Titulação do VDRL do parto				0,004
	≤ 1:8	344 (72,0)	1	
	> 1:8	134 (28,0)	2,46 (1,33–4,53)	
Tratamento da gestante no pré-natal				< 0,001
	Com penicilina benzatina	180 (45,4)	1	
	Outra droga/não tratada	216 (54,6)	3,52 (1,74–7,13)	

## DISCUSSÃO

Neste estudo, encontrou-se desfecho de prematuridade em 15,3% dos casos de SC, porcentagem superior às taxas estimadas para gestantes em geral descritas em estudo no Brasil: entre 7,7%^16^ e 11,1%^17^. Gestantes que durante o pré-natal não foram tratadas ou receberam tratamento com drogas diferentes da penicilina benzatina, bem como as que apresentaram elevados títulos de VDRL no parto, tiveram mais desfecho de prematuridade.

Pode-se observar que uma quantidade significativa de gestantes com sífilis que tiveram bebês prematuros frequentou o pré-natal. Contudo, muitas oportunidades para prevenir a SC foram perdidas, situação evidenciada também em outro estudo, realizado em seis capitais brasileiras^18^. O cuidado pré-natal pode impactar positivamente a saúde da gestante e evitar a mortalidade infantil^19^, e é um importante preditor para prevenir desfechos desfavoráveis relacionados à sífilis na gestação, desde que realizado com qualidade^20^^,^^21^.

Dentre a perda de oportunidades, destacam-se a falta de diagnóstico e falhas no tratamento das gestantes. Estudo de metanálise mostrou que as intervenções no pré-natal diminuem significativamente o risco de a gestante ter um resultado adverso devido à sífilis^22^. No entanto, no período deste estudo, o exame de rotina solicitado às gestantes era o VDRL, não havia coleta diária de sangue nas unidades, e o retorno dos resultados estava disponível somente 15 dias após a coleta, dificultando o acesso ao diagnóstico. Ademais, o teste rápido (TR) se encontrava em fase de implantação.

O TR para sífilis é uma estratégia que pode contribuir para aumentar a cobertura de testagem em gestantes, especialmente quando realizado na primeira consulta, possibilitando o tratamento imediato. Considerando que a grande maioria das gestantes recebeu duas ou mais consultas e iniciou o pré-natal antes do final do segundo trimestre de gestação, haveria tempo hábil para tratamento adequado caso tivessem realizado o TR.

Ocorre que, durante o período de realização deste estudo, o Brasil vivenciou um problema grave de escassez de penicilina^23^, situação que contribuiu para que muitas gestantes não fossem tratadas ou recebessem medicamentos diferentes da penicilina benzatina. Estas gestantes apresentaram 3,52 vezes mais chances de terem bebês prematuros, situação também evidenciada em outros estudos realizados na China, que identificaram maiores chances de prematuridade e de outros desfechos em gestantes com sífilis não tratadas ou inadequadamente tratadas^24^^,^^25^.

A possibilidade de recorrência de escassez de penicilinas^26^ é preocupante, visto que a penicilina benzatina é a droga de escolha para tratamento da sífilis em gestantes por ser a única que atravessa a barreira transplacentária e trata o bebê^23^. Por esse motivo, é necessário desenvolver estudos que avaliem a eficácia de drogas alternativas^27^.

Nesse estudo, também foi identificado maior chance de prematuridade em gestantes cuja titulação do VDRL foi > 1:8 na ocasião do parto. Altos títulos de VDRL em gestantes durante o pré-natal e no momento do parto podem representar sífilis ativa, condição associada a desfechos graves de SC, dentre eles a prematuridade^3^^,^^28^. Por esse motivo, após o tratamento, o controle de cura da sífilis em gestantes deve ocorrer mensalmente, por meio do monitoramento da queda dos títulos do teste VDRL^15^.

Uma análise detalhada dos casos de gestantes que apresentaram altas titulações do VDRL na ocasião do parto mostrou que nenhuma mulher que recebeu tratamento completo (três doses de penicilina benzatina) teve bebê prematuro, reforçando a importância do diagnóstico e do tratamento de gestantes com sífilis em tempo hábil, prevenindo desfechos graves, como a prematuridade em decorrência da SC.

Este estudo tem como limitação o fato de ter analisado dados secundários, considerando que a falta de registro ou a baixa qualidade dos dados são comuns. Incluir a coleta em diferentes fontes de dados (fichas de notificação e prontuários) contribuiu sobremaneira para minimizar o problema. Ademais, a exclusão dos casos de natimortos, devido à falta de registro de variáveis importantes, e de gestantes que apresentavam agravos que pudessem interferir no desfecho de prematuridade por SC, podem ter subestimado o número de prematuros.

Os achados deste estudo evidenciam que a prematuridade decorrente da SC é evitável, desde que as gestantes com sífilis sejam tratadas adequadamente. Identificou-se também que fragilidades na assistência pré-natal estão associadas a este desfecho, o que ressalta a importância de implementar políticas públicas para melhorar a qualidade do pré-natal.

Nesse sentido, a atenção primária em saúde tem papel imprescindível para detectar a sífilis em gestantes e garantir a realização de exames e do tratamento. Esses esforços para prevenir a prematuridade por SC podem evitar repercussões importantes para o bebê, para a família e para o sistema de saúde.
